# The effect of color coding and layout coding on users’ visual search on mobile map navigation icons

**DOI:** 10.3389/fpsyg.2022.1040533

**Published:** 2022-12-13

**Authors:** Mengzhe Zhang, Yong Gong, Rong Deng, Sanyuan Zhang

**Affiliations:** ^1^Pan Tianshou College of Architecture, Arts and Design, Ningbo University, Ningbo, China; ^2^School of Design, Jiangnan University, Wuxi, China; ^3^College of Computer Science and Technology, Zhejiang University, Hangzhou, China

**Keywords:** color coding, spatial layout, mobile interface, navigation icons, visual search, eye tracking

## Abstract

Color and spatial layout are important factors that affect users’ icon cognition and play a huge role in the visual search process of icons. Guided by the user’s interactive needs, this paper aims to improve the visual search efficiency of mobile map navigation icons. The mixed design within and between subjects is adopted through the combination of theoretical and experimental research, and the subjective questionnaire method is used to explore the research. This paper explores the visual search problem of mobile map navigation icons based on color coding and layout coding. The experimental results mainly include reaction time, accuracy rate, user experience, and statistical and variance analysis. The results show that the layout of the mobile map navigation icons significantly impacts the user’s visual search. The navigation icons that use color for layout coding have the highest visual search efficiency and better user experience. Among the icons, the layout with regular color distribution and a larger area of the same color has the highest visual search efficiency for users and the best user experience; the visual search efficiency of navigation icons using color for layout coding is significantly higher than that of mobile map navigation icons.

**Relevance to industry:** The user scale of mobile information maps is huge and the usage rate is high, but the large number of navigation icons increases the burden of user information identification and acquisition. As a result, the efficiency of user information acquisition is low, and the user experience is reduced. A clear, easy-to-search navigation icon design can enhance the user experience of the entire product. The results of this research provide theoretical support and practical guidance for the design optimization and improvement of mobile map navigation icons.

## Introduction

The development and popularization of network communication technology have facilitated the life and travel of users. With the development of science and technology, the development of mobile terminals, and the maturity of 4G and 5G, the mobile map has developed rapidly. In 2008, policies related to mobile maps were introduced, which brought the industry into a period of rapid development. In 2021, the number of mobile map users in China will reach 800 million (IIMedia Research Center for Life and Travel Industry, 2019). The mobile map has the characteristics of easy portability, accurate positioning, and comprehensive data. It plays an important role in people’s travel and has a broad market and far-reaching influence.

People obtain services through mobile information maps, such as route navigation, real-time positioning, location search, location sharing, and surrounding discovery. This information is presented in the navigation system of the mobile map in the form of icons. Icons can quickly transmit information, highly condense information, and facilitate information memory. The good design and application of navigation icons greatly help to improve users’ visual search efficiency and experience when using information maps on mobile terminals. Recognition and recognition of navigation icons rely on visual search, users’ visual search for navigation icons directly affects the efficiency of using mobile maps. Visual search is a complex cognitive process and is a common experimental paradigm for studying visual perception processing and attention mechanisms. Visual search is inseparable from attentional processing. The main factors affecting attentional processing are object features and locations. Therefore, feature attention and spatial attention are important factors affecting visual search. Only by refining and structuring the navigation icons of the mobile map in terms of features and spatial layout can the user’s visual search efficiency for navigation icons be improved, and it is convenient for people to browse and understand quickly and easily. At the same time, the user experience of the mobile map is improved.

### Visual search for icon colors

Color plays a very obvious role in the various factors that affect visual search. As the most direct and effective coding method, the first thing users see is color information. Designers use different colors to categorize interface information to help people find icons and buttons. Consistent and easy-to-understand color coding can help users easily understand their internal connections and help them search more accurately (Steven, 2008). Preferential assignment of colors by vision relies on selective processing of attention (Li and Xue, 2016). When users search for icons, they will first classify different icons by vision, code them according to color, then pay attention to processing to filter and layer the icons, and finally, pay attention to in-depth processing to focus on the target. Research on attentional capture of color has shown that users will have a layered effect on visual perception in the process of color coding, which affects the attention order of information and search performance (Li et al., 2018). Users will have a psychological sense of advance and retreat for different colors, then form different perception depths, that is, color perception distances, leading to visual perception stratification. Color coding can guide the user’s visual behavior in the icon search process, and users can effectively improve cognitive performance by color coding through visual perception hierarchy. The visual layering method was proposed by Laar (2001a,b) in the study of color-enhancing visual display design. The research on color perception distance believes that the three attributes of lightness, hue and saturation (Munsell color stereo) are in line with the three-dimensional equidistance of people’s vision. Therefore, the same perceived distance is one of the variables to be controlled when selecting the icon color. The CIE L*a*b color space is the most comprehensive representation of the spectrum seen by the human eye and provides a reference for most common color spaces. Therefore, the icon colors in the experimental materials should use the colors with the same perceptual distance in the CIE Lab space.

Studies have examined the effect of display chromaticity contrast on multicolor GUIs (Bodrogi, 2003). The research on the color change of interactive elements by the eye control system verifies that color saturation and brightness can affect the users’ visual search efficiency (Niu et al., 2022). Related research on factors such as different background color classification of icons and color combinations between icons and backgrounds have found that color significantly impacts icon visual search. Color features play an important role in visual pre-attention processing. The smaller the icon background area divided by color, the lower the users’ visual search efficiency (Michalski and Grobelny, 2008; Michalski, 2014). In addition to the research on the icons’ background color, the related research on the color of the icon itself and other physical properties has confirmed that color coding is the most effective coding method for the usability of the icon. The effectiveness of shape coding is higher than that of icon size (Nowell, 1997). The research on color perception distance believes that the three attributes of lightness, hue, and saturation (Munsell color stereo) align with the three-dimensional equidistance of users’ vision. A study was conducted on users’ icon cognition through attributes such as hue, saturation, and brightness, and it was found that color significantly impacted users’ visual search (Hsieh, 2017). Dennis (2008) pointed out through research that using color attributes such as lightness, hue, and saturation to design relevant information can help users identify information and improve search efficiency (Huang, 2008). Wu and Chen (2009) studied 18 GUIs containing color attributes of sofa appearance and found that color grouping was one of the most important factors affecting users’ performance and satisfaction. Previous studies have studied the effect of icon and background color combination and icon and background area ratio on visual search efficiency. The research found that the color combination significantly impacts the icons’ visual search efficiency. The visual search efficiency of the icon with black blue and black yellow color matching is higher than white-yellow or white-blue, and the visual search efficiency of an icon with an area ratio of 90% to the background is higher than that of an icon with an area ratio of 70% (Huang, 2008). The consistency and number of icon colors will affect the visual search of icons. Increasing the number of colors will reduce the visual search efficiency of icons to a certain extent. Using colors to increase the difference between icons can effectively improve the visual search efficiency of icons (Gong et al., 2016a). The research found that the shape of smartphone application icons has no significant impact on visual search; the color contrast significantly impacts icon visual search, and low-contrast icons can attract users’ attention more than high-contrast icons. Icons are more efficient for visual search than icons combined with graphic and text patterns (Jiang et al., 2015). In smartphone interfaces, research on the search efficiency of icon colors and borders shows that icons’ different colors and rounded square borders can improve users’ visual search efficiency and reduce cognitive load (Liu et al., 2021). In the process of complex information transmission, color plays a huge role in visual perception and cognition, and color coding is an important information coding method of visual search. Therefore, it is significant to study the effect of color coding on the interaction interface on the visual search of user icons. It is necessary to further explore how users search for navigation icons of different colors when using mobile phone maps.

### Visual search for icon space layout

Spatial layout plays an important role in visual search, and interface icon design is inseparable from the spatial layout design. The layout of icons refers to the arrangement and design of related elements such as icons in the interface. By arranging and designing the position, color, and size of the icons in the interface, it helps users to quickly find the target content on the interface (Peng, 2021). The spatial layout design of the human-computer interaction interface is mainly to regularize and organize the information and establish a visual structure. The information processing capability of the human visual system is limited, and the layout of interface information has a huge impact on the user’s visual search efficiency (Wolfe and Horowitz, 2007). In the human-computer interaction interface, the perceptual sequence plays an important role in guiding the information encoding of the brain, and a good information layout and visualization structure can reduce the occurrence of cognitive load problems of users (Li, 2018). The brain processing of perceptual order is based on the principle of spatiotemporal proximity. Users can associate visual elements acquired at different times to form spatial structures according to the sequence, location, interval and other attributes of interface information, and perform cognitive processing on different structures according to the time sequence (Amiez and Petrides, 2007). The information coding basis of perceptual order is the principle of spatial configuration. The spatial configuration is the input of information direction and position, and the output of information association based on information and spatial structure characteristics (Li, 2018). According to the principle of space configuration, the layout design and visual structure design of icons can help users to configure the interface icons. From the above research, it is not difficult to find that most of the research on the effect of color on the visual search of icons is related to the spatial layout. And the results show that feature attention and spatial in visual search are carried out simultaneously.

The related research on icon grouping layout design and visual search shows that the display method of classifying icons can significantly improve visual search efficiency (Niemelä and Saarinen, 2000). The experimental results combined with eye movement features show that grouped icons have fewer fixations than randomly arranged icons, and users of grouped icons have higher visual search efficiency, further confirming this result (Murata and Furukawa, 2005). Fleetwood et al. used the adaptive control of thought-rational/perceptual motor (ACT-R/PM) model of the adaptive control system, combined with eye movement experiments to study the users’ visual search strategy. They examined the influence of the icon frame, icon quality, text and other factors on the visual search efficiency, and the results found that when searching for high-quality icons in interference icons, the subjects will use the group search strategy; when searching for low-quality icons, the subjects will change the search Strategies are searched by text (Fleetwood and Byrne, 2002, 2006). Spatial layout factors affecting icons’ visual search include color matching, arrangement, and target location. The eye smooth pursuit study by Euclidean algorithm pointed out that the variable in the center position has the greatest influence on the tracking efficiency (Niu et al., 2021). When the navigation panel is located in different positions on the screen, it will impact the users’ visual search performance. When the navigation panel is located at the top or left of the screen, the users’ search efficiency is more efficient (Schaik and Lin, 2001). In a similar study, through the study of different positions of the icon panel, it was found that when the panel is located on the left, right, or top and bottom of the interface, there is no significant difference in the users’ search efficiency (Pearson and Schaik, 2003). In the experiment on the number of icons, arrangement, icon panel structure, and other factors affecting the users’ response time, it is found that the user operation efficiency of the compact square structure icon panel is the highest, followed by the horizontal arrangement, and finally the vertical arrangement (Grobelny et al., 2005). Based on previous research, Michalski et al. found that when the icons are arranged vertically, they is located on the screens’ left or right side, does not significantly impact the users’ visual search time (Michalski et al., 2006). A recent study by domestic scholars examined the effect of different icon structures and positions on user search efficiency. The results found that the icons with a square layout have the highest operating efficiency for users, while the circular layout has the lowest operational efficiency. There is no significant difference between icons’ horizontal and vertical distribution (Chen and Chiang, 2011). Further combining the eye movement experiment and the ACT-R model, through the analysis of eye movement data such as eye movement trajectory and visual heat map, it is found that there is also a relationship between color and shape and visual search. Users will first use color during the search process to implement a ‘grouping strategy’ to improve search efficiency (Wang et al., 2016). Gong et al. (2016a) investigated the influence of factors such as icon size, icon panel distribution direction, layout, and icon location on users’ visual search. The results showed that the smaller the icon, the lower the users’ search efficiency. The larger the aspect ratio of the icon panel, the lower the search efficiency. When the icon is located at the top of the screen, the users’ visual search efficiency is higher than that at the bottom. The visual search efficiency of the horizontally distributed icons is higher than that of the vertically distributed icons. The difference in search efficiency between the horizontal and vertical distribution of icons varies with the aspect ratio value of the icon panel (Gong et al., 2013). Different layout structures of icons affect the users’ visual search efficiency. The search efficiency is the lowest when the icon layout structure is annular. When the icon layout structure is rectangular or round, there is no significant difference in the users’ visual search efficiency (Kong, 2021). The visual characteristics and number of icons also affect the layout of icons. When the number of icons is 6, the visual characteristics of icons are the main factors affecting users’ visual search. When the number of icons is 15, the layout is the main factor affecting visual search, and the horizontal Distributed icons are most efficient for visual search (Jin et al., 2021). The above research shows that the spatial layout of icons through grouping and other means can effectively suppress the interference of non-target stimuli, thereby improving the visual search efficiency of icons.

Mobile phone map navigation icons are usually presented in a random arrangement of 3–4 colors. How different layouts affect users’ visual search needs further analysis.

### Purpose of the study

At this stage, the problems related to the presentation and visualization of the navigation icon interface of the mobile map are discussed more from the product itself. There are few pieces of research on the visual design of the mobile map, and there are very few interface designs specifically for navigation icons.

This research will analyze the navigation icons of the mobile map, use the selective color simplification method to process the icons, and design and carry out the visual search experiment of the icons under the user’s color coding and different spatial layout combination designs. At the same time, the research investigates the subjects’ subjective feelings, studies the visual search efficiency of users under different colors and layouts, and compares the visual search levels of users under different color coding. Finally, this paper will propose the color and layout scheme of the best mobile map navigation icons based on visual search and provide a scientific basis for the design of mobile terminal navigation icons.

## Materials and methods

### Experiment design

The experiment adopts the experimental method of within-subject and between-subject mixed design. The research includes two independent variables, color (R, G, B) and grouping method (G0 is a single color without grouping, G1 is a random distribution grouping method, G2 (1 × 5), G3 (3 × 5)), color is a between-subject variable, and grouping method is a within-subject variable. Therefore, this is a 3 × 4 mixed design experiment. The experiment includes six experimental conditions, R, G, B, G1, G2, and G3, of which R, G, and B are all grouped by G0 (as shown in Table 1). The experiment was conducted in three groups, and each participant completed one of the tasks. Under any experimental condition, subjects need to complete 10 search tasks. In order to increase the randomness during the experiment, the target will not appear in 3 search tasks. Therefore, each subject needs to complete 40 visual search tasks.

**Table 1:** Six experimental conditions and their layout.

Serial number	Color	Layout	Icon Form	G1	G2	G3
1	R	-	G0	*(1)		
2	G	-	G0		*(1)	
3	B	-	G0			*(1)
4	-	Random	G1	*(2)	*(2)	*(2)
5	-	1*5	G2	*(3)	*(3)	*(3)
6	-	3*5	G3	*(4)	*(4)	*(4)

*represents the number of experiments under this test condition.

### Experiment material

**Figure 1:**
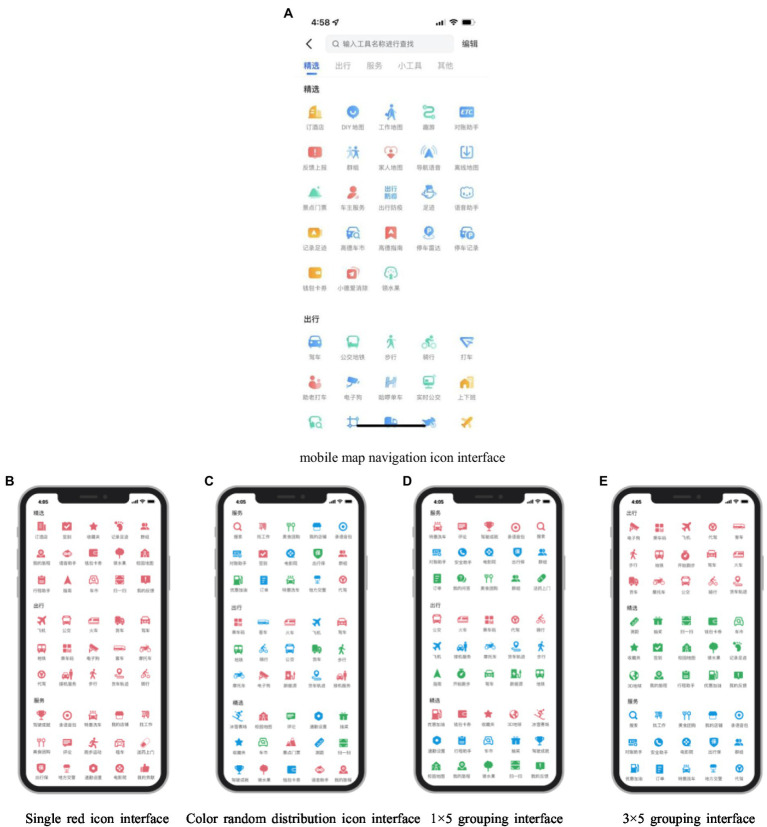
The icon interface used in the experiment. **(A)** mobile map navigation icon interface. **(B)** Single red icon interface **(C)** Color random distribution icon interface **(D)** 1 × 5 grouping interface **(E)** 3 × 5 grouping interface.

In the experiment, the CIE Lab color space is used as a color coding tool. In the CIE Lab color space, red (R), green (G), and blue (B) with the same perceptual distance are selected as the icon colors. The specific color specifications are shown in Table 2. In order to make the experiment more in line with the real icon search situation, this experiment selected a representative mobile map - AutoNavi map navigation icon interface for design, including icon content (with AutoNavi map navigation icon as reference), icon type (Select the flat icons used in the mobile map navigation icons) and the icon arrangement (5 icons in a row) to ensure that the display effect of the icons seen by the subjects in the experiment is the same as the presentation effect of the real mobile map navigation icons. The experimental materials are presented together with icons and text, and the way in which the experimental materials are presented will not affect the subjects. Zhang Yue studied the visual cognition of the design of the APP interface navigation system and found that when the text is used as auxiliary information next to the icon, the user can directly identify it through the icon, and the auxiliary text is mostly ignored by the user (Zhang, 2017). In order to ensure the randomness of the experiment, the design of monochrome mobile map navigation icons is retained in this experiment. Figure 1A is the current mobile map navigation icon interface, Figure 1B is the single-color icon interface G0, and Figure 1C is the three-color combination randomly distributed icon interface G1 (in this experiment, the existing mobile map navigation icon interface), Figure 1D is the 1 × 5 layout icon interface G2, Figure 1E is the 3 × 5 layout icon interface G3.

**Table 2:** Colors used in icons.

Color	Color sample	CIE LAB	Decimal RGB	Color pantone
R	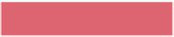	(58, 49, 17)	(221, 101, 114)	# DD 65 72
G	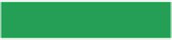	(58, −47, 29)	(36, 159, 85)	# 24 9F 55
B	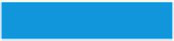	(58, −14, −45)	(18, 150, 219)	# 12 96 DB

### Experiment equipment

**Figure 2:**
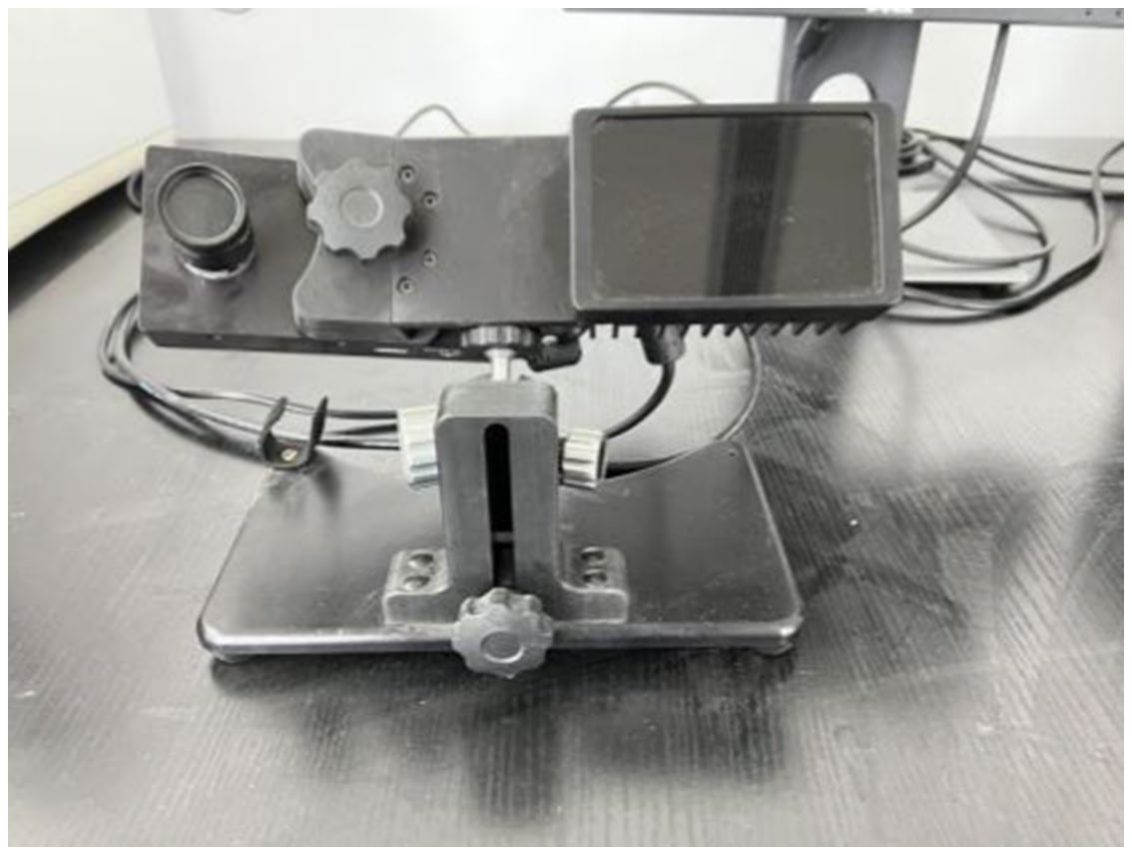
EyeLink1000 Desktop Eye Tracker.

**Figure 3:**
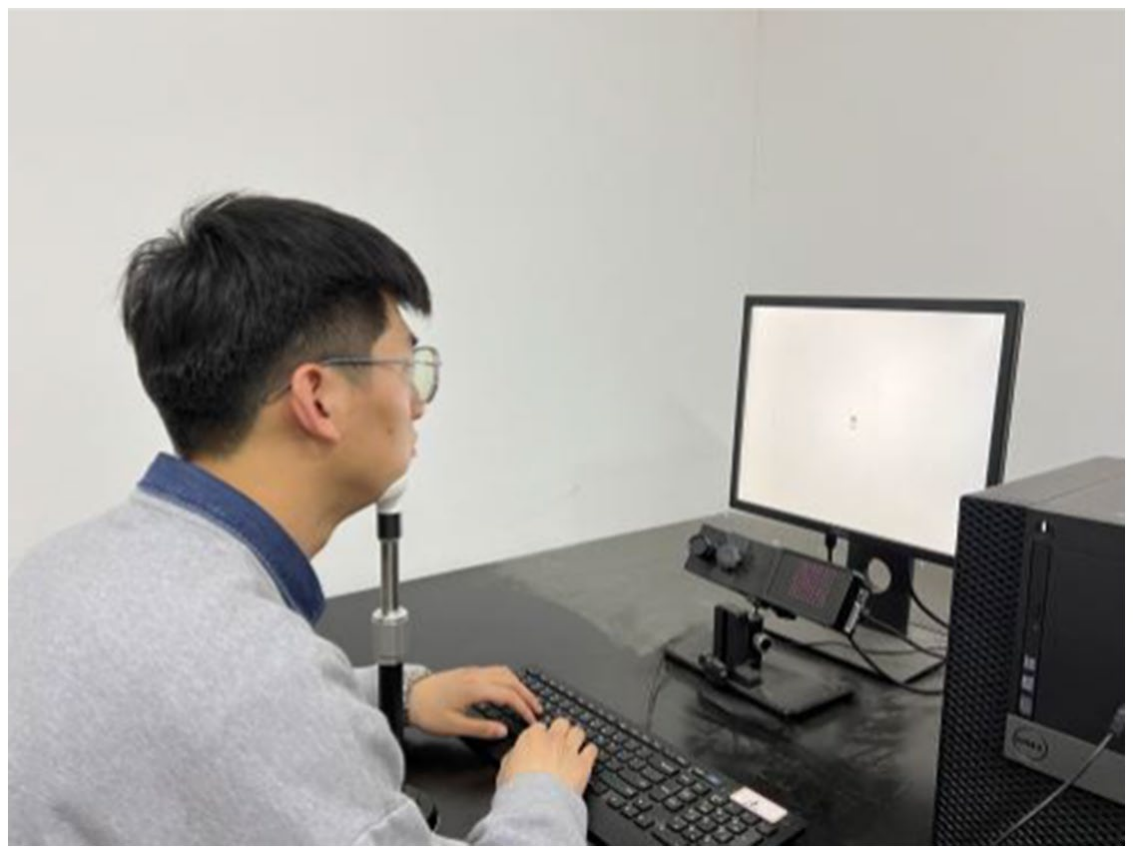
Photo of participant participating in the experiment.

The experiment was written and controlled through the Experiment Builder program, and the behavioral data of the subjects were automatically recorded in a text file. The eye tracking instrument is the EyeLink1000 desktop eye tracker (as shown in Figure 2) produced by SR Company in Canada, and the sampling frequency of the eye tracker is 1,000 times/s. Stimulus material was displayed in the center of a 19-inch Dell computer monitor with a resolution of 1,024 × 768 pixels and a refresh rate of 100 Hz. The distance between the subjects’ eyes and the computer screen was about 50 cm. The subjects completed the experimental tasks by pressing the keys on the keyboard. Figure 3 was taken during the operation of the tested experiment.

### Participants

The experimental subjects were 30 undergraduate and postgraduate students, including 13 boys and 17 girls, aged 21–29. All subjects were right-handed with normal or corrected-to-normal vision and no color blindness or color weakness. All the subjects have not done similar experiments before, and they can get a certain reward after the experiment.

### Procedure

**Figure 4:**
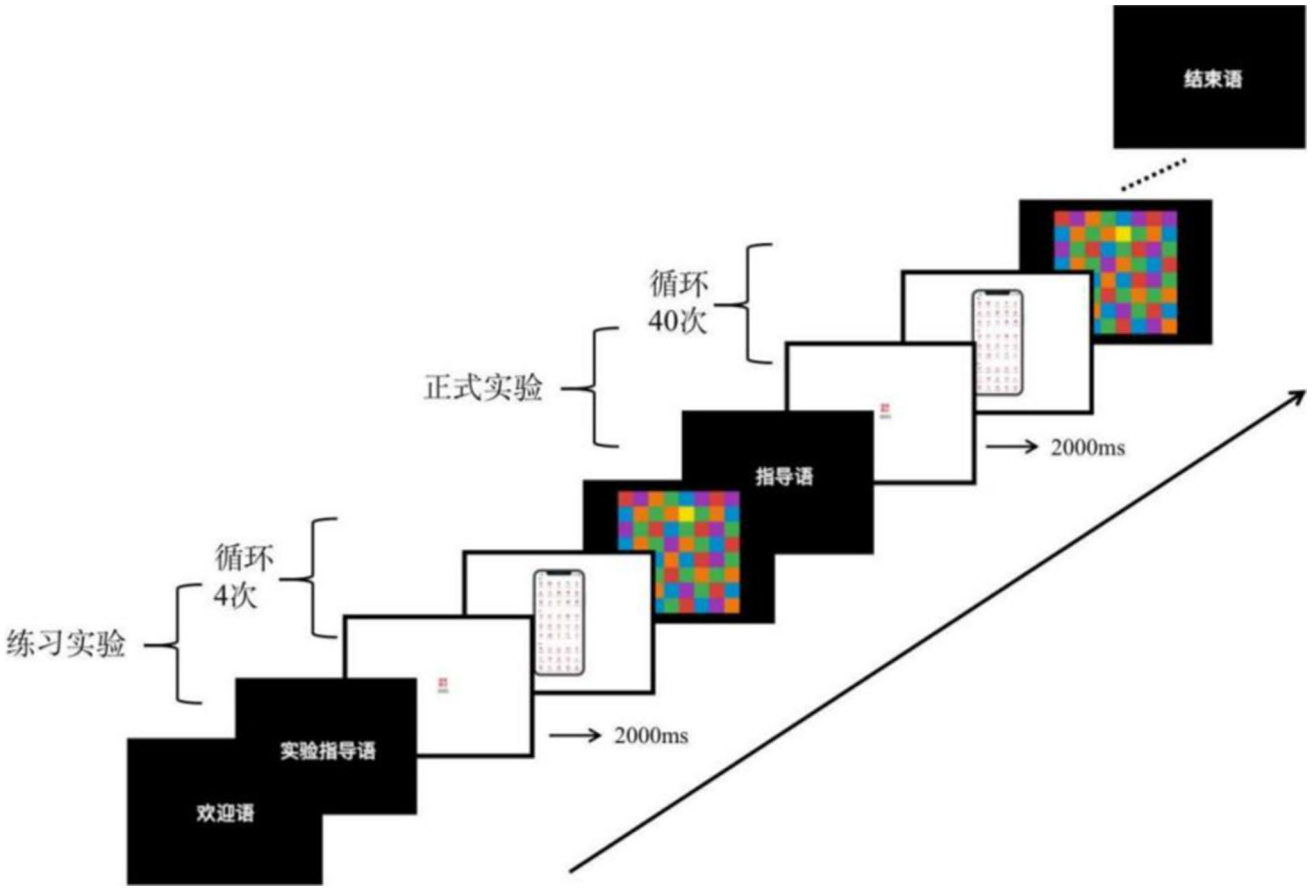
Experimental procedure example.

The whole experiment is divided into two parts, the first part is a behavioral experiment and eye movement experiment, and the second part is a subjective questionnaire. Behavioral and eye-movement experiments were conducted on an individual basis. After the subjects entered the laboratory, they first sat in front of the test machine with their eyes perpendicular to the screen and then supported their chins on the fixed bracket to keep their heads still to ensure the accurate tracking of the eyes by the experimental equipment. Before the start of the normal laboratory, the first is to conduct a practice experiment. The main tester presents the experimental instructions to the subjects through the monitor to ensure that the subjects understand the experimental procedures. Then perform eye calibration through the eye tracker, and then start the practice experiment. The practice experiment performs four keystroke responses. The operation of the practice experiment is consistent with the formal experiment to ensure that the subjects are proficient in key-pressing tasks and reduce experimental errors. After completing the practice experiment, the subjects rested for 10 s and then started the formal experiment. The first target icon is displayed in the center of the screen, the icon disappears after 2000 ms, and the navigation icon interface under a certain experimental condition is displayed in the center of the screen. The subject needs to search for the target icon that appeared before in the interface. If the target icon is found, press the “f” key; if not found, press the “j” key. After pressing the button, the masking stimulus is presented in the center of the screen, and the experiment ends here. At this point, a search task is completed. A random screen displays the target icon for the second search mission, which starts. This is done in sequence until the subjects complete all the search tasks. Figure 4 shows an example of the experimental process.

The subjective questionnaire includes three dimensions, satisfaction, usability, and preference. The questionnaire adopts the 9-level Likert scale evaluation method, and there are 15 questions on all the scales.

## Results

The experimental results include behavioral data and eye movement data. The behavioral data includes reaction time and accuracy, and the eye movement data includes the number of gazes, Gaze time, and saccade length. Behavioral data were recorded by EB, and eye movement data were recorded and exported by DateViewer. The behavioral and eye movement data were processed and analyzed by SPSS.

### Behavioral experimental data

The experimental behavioral data of the subjects under the six experimental conditions are counted, and the specific conditions are shown in Table 3.

**Table 3:** Behavioral data for six experimental conditions.

Color	Layout	Average response time/ms		Average correct rate/%	
		Average	Standard deviation	Average	Standard deviation
R	G0	3,678.577	1,078.053	88.57	1.127
G		4,257.810	1,227.204	90.00	1.355
B		3,086.516	1,495.224	80.00	1.205
	G1	2,958.876	909.632	85.71	1.353
	G2	2,105.345	466.730	92.38	1.109
	G3	2,272.131	575.173	90.95	1.155

Repeated measures ANOVA was performed on response time, and the results given in Table 4 shows that the main effect of layout was very significant, *F*(2, 54) = 35.409, *p* < 0.05; the main effect of color was not significant, *F*(2, 54) = 0.717, *p* = 0.497; the interaction of color and layout was not significant, *F*(4, 54) = 1.540, *p* = 0.102. For further analysis of the layout, the pairwise comparison results showed that the difference between G1 and G2 was significant (*p* < 0.001), the difference between G1 and G3 was significant (*p* < 0.001), and the difference between G2 and G3 was not significant (*p* < 0.001) = 0.332). This result shows that color is not the main factor affecting the visual search efficiency of icons, and the spatial layout design can significantly improve the visual search efficiency of users. Since the monochrome navigation icon interface does not belong to any layout method, the monochrome navigation icon interface G0 is not involved in the previous pairwise comparison.

**Table 4:** Results of repeated measures ANOVA on visual search time.

Factor	df	*F*	*p*
Layout	2	35.409	0.000
Color	2	0.717	0.497
Layout × color	4	1.540	0.204

*p < 0.05. *Based on the estimated marginal mean. The mean difference was significant at 0.05 level. 95% confidence interval.

In order to further explore the influence of different layout methods on the visual search efficiency of icons, a one-way analysis of variance was performed on the response time data of 4 different layout methods. The results are shown in Table 5, the difference between G0 and G2 was significant (*p* < 0.001), and the difference between G0 and G3 was significant (*p* < 0.001); the difference between G1 and G2 was significant (*p* < 0.001), and the difference between G1 and G3 was significant (*p* = 0.006).); the difference between G2 and G3 was not significant (*p* = 0.769). The result shows that, compared with the monochromatic navigation icon interface, the icon interface using color for layout coding has higher visual search efficiency. The visual search efficiency of the layout method with random color distribution is not significantly different from that of the monochrome icon interface, but the visual search efficiency of the layout method with more regular color distribution (G2, G3) is significantly higher than that of the random distribution layout method.

**Table 5:** One-way ANOVA test for different layouts (reaction time).

	G0	G1	G2	G3
G0	×	0.103	0.000	0.000
G1		×	0.000	0.006
G2			×	0.769
G3				×

*p < 0.05. *Based on the estimated marginal mean. The mean difference was significant at 0.05 level. 95% confidence interval.

Repeated-measures ANOVA was performed at the correct rate, and the results as Table 6. showed that the main effect of the layout was significant, *F*(2, 54) = 3.578, *p* = 0.035, indicating differences among different layouts of G1, G2, and G3. The interaction between color and layout is not significant, *F* < 1. The output of the between-subject variable showed that the main effect of color was not significant, *F*(2, 54) = 1.056, *p* = 0.362. The generalized linear model analysis of the layout method shows that the difference between G1 and G2 is significant (*p* = 0.030), the difference between G1 and G3 is not significant (*p* = 0.088), and the difference between G2 and G3 is not significant (*p* = 0.642). This result shows that the search accuracy rate of the subjects is not affected by color, but there are significant differences in the layout. The icon interface G1 with random color distribution has the lowest search accuracy rate of 85.71%, and the color distribution regular G2 and G3 grouping search. The correct rate is higher than that of G1, and the correct rate of G3 is higher than that of G2, indicating that in different layout methods, the grouping arrangement (G3) with a larger area of the same color has a higher search accuracy.

**Table 6:** Results of repeated measures ANOVA on correct rate.

Factor	df	*F*	*p*
Layout	2	3.578	0.035
Color	2	1.056	0.362
Layout × color	4	0.845	0.503

*p < 0.05. *Based on the estimated marginal mean. The mean difference was significant at 0.05 level. 95% confidence interval.

Further one-way ANOVA was performed on the correct rates of the four different layout methods. The results are shown in Table 7, the difference between G0 and G2 is marginally significant (*p* = 0.053), and the difference between G0 and the other two layout methods, G1 and G3, is not significant (*p* > 0.05); the difference between G1 and G2 Significant (*p* = 0.037), the difference between G1 and G3 was not significant (*p* = 0.101); the difference between G2 and G3 was not significant (*p* = 0.653). The result shows that for the correct rate, there are differences between G2 with regular color distribution, G0 with monochrome navigation icon interface, and G1 with the random color distribution. Among the icons with regular color distribution, the icon layout (G3) with a larger area of the same color has a higher visual search accuracy.

**Table 7:** One-way ANOVA test for different layouts (correct rate).

	G0	G1	G2	G3
G0	×	0.881	0.053	0.135
G1		×	0.037	0.101
G2			×	0.653
G3				×

*p < 0.05. *Based on the estimated marginal mean. The mean difference was significant at 0.05 level. 95% confidence interval.

### Eye movement data

Table 8 shows the eye movement data of the subjects under the six experimental conditions, including the number of fixations, fixation time, saccade length, and their mean and standard deviation.

**Table 8:** Eye movement data for six experimental conditions.

Color	Layout	Number of fixations/time		Fixations time/ms		Saccade length/rad	
		Average	Standard deviation	Average	Standard deviation	Average	Standard deviation
R	G0	20.363	5.301	5,316.125	1,605.910	46.749	16.040
G		21.410	3.756	5,612.895	1,013.379	51.371	10.823
B		17.355	6.147	4,725.907	1,329.726	41.167	14.737
	G1	16.296	3.603	4,421.482	875.517	36.022	11.172
	G2	14.233	3.529	3,953.692	742.815	27.417	10.260
	G3	14.124	2.718	4,077.282	974.845	29.728	7.453

The number of fixations is the number of all the fixation points of the subjects during the visual search during the period of interest and the area of interest. The number of fixation points can indicate the depth of information processing by the subjects during visual search. In this experiment, the more the number of fixations, the deeper the information processing, and the lower the search efficiency. Through repeated measures ANOVA on number of fixations (Table 9), it was found that the main effect of grouping mode was very significant, *F*(2, 54) = 10.106, *p* < 0.01. The interaction between color and different layouts was not significant, *F*(4, 54) = 1.044, *p* > 0.05. The output of the between-subject variable showed that the main effect of color was not significant, *F*(2, 54) < 1, *p* = 0.528. Further generalized linear model analysis was conducted on different layouts of navigation icons. The results showed that the number of fixations between G1 and G2 was significantly different (*p* = 0.016), the number of fixations between G1 and G3 was significantly different (*p* = 0.011), and the number of fixations between G1 and G3 was significant (p = 0.011). The difference in the number of fixations with G3 was not significant (*p* = 0.899). The result shows that in the mobile map navigation icon interface, when users search for icons, the color has no significant impact on the number of gazes, and the layout significantly affects the number of fixations when users search for icons. And the number of fixations of the subjects in the navigation icon interface with regular color distribution is less than that of icons with a random color distribution.

**Table 9:** Results of repeated measures ANOVA on number of fixations.

Factor	df	*F*	*p*
Layout	2	10.106	0.000
Color	2	0.654	0.528
Layout × color	4	1.044	0.393

*p < 0.05. *Based on the estimated marginal mean. The mean difference was significant at 0.05 level. 95% confidence interval.

In order to further explore the influence of different layout methods on the number of subjects’ fixations, a one-way analysis of variance was performed on the four different layout methods. The results are shown in Table 10. The difference between G0 and G1 was significant (*p* = 0.040), the difference between G0 and G2 was significant (*p* < 0.001), and the difference between G0 and G3 was significant (*p* < 0.001), the difference among G1, G2, G3 was not significant (*p* > 0.05). The result shows that the number of fixations of monochrome navigation icons is more than that of navigation icons laid out by color. There is no significant difference in the number of fixations between navigation icons laid out by color.

**Table 10:** One-way ANOVA test for different layouts (number of fixations).

	G0	G1	G2	G3
G0	×	0.040	0.000	0.000
G1		×	0.172	0.070
G2			×	1.000
G3				×

*p < 0.05. *Based on the estimated marginal mean. The mean difference was significant at 0.05 level. 95% confidence interval.

The fixation time is the sum of the durations of all fixation points in the period or area from the time the subject’s fixation point enters the interest period or area of interest until the fixation point leaves the period or area. The fixation time indicates the time spent by the subjects in visual search in a certain period or area. In this experiment, the longer the subject’s fixation time, the more time the subject spends on the search task, and the lower the visual search efficiency. Using repeated measures ANOVA to analyze fixation time, the results (as Table 11) showed that the main effect of the layout was significant, *F*(2, 54) = 4.893, *p* = 0.011. The interaction between color and layout was insignificant, *F* < 1, *p* > 0.05. The output of the between-subject variable showed that the main effect of color was not significant, *F*(2, 54) = 0.417, *p* = 0.663. Further pairwise comparison of different layouts shows that the difference between G1 and G2 is significant (*p* = 0.037), the difference between G1 and G3 is not significant (*p* = 0.125), and the difference between G2 and G3 is not significant (*p* = 0.125). 0.582). The result shows that the fixation time of the navigation icons with random color distribution is higher than that of the navigation icon interface with a regular color distribution. In the navigation icon interface with regular color distribution, subjects in the same layout with a larger color distribution area, their fixation time is shorter than in the same layout with a smaller color distribution area.

**Table 11:** Results of repeated measures ANOVA on fixation time.

Factor	df	*F*	*p*
Layout	2	4.893	0.011
Color	2	0.417	0.663
Layout × color	4	0.797	0.532

*p < 0.05. *Based on the estimated marginal mean. The mean difference was significant at 0.05 level. 95% confidence interval.

Further one-way analysis of variance was performed on the four different layout methods, and it was found that the effect of layout methods on fixation time was consistent with the analysis results of fixation times. The difference between G0 and G1 was significant (*p* = 0.040), the difference between G0 and G2 was significant (*p* < 0.001), and the difference between G0 and G3 was significant (*p* < 0.001); The difference was not significant (*p* > 0.05). The result shows that the fixation time of monochrome navigation icons is more than that of navigation icon interfaces that use color for layout coding. There is no significant difference in fixation time between navigation icons laid out by color coding.

The saccade length is the sum of all distances from the gaze point to different positions in a certain interest period or area of interest. In this experiment, the longer the saccade length, the more disordered the subjects’ gaze in the search task, and the lower the visual search efficiency. Repeated measures ANOVA of saccade length (as Table 12) showed that the main effect of layout was significant, *F*(2, 54) = 12.928, *p* < 0.05. The interaction between layout and color was not significant, *F*(4, 54) = 1.313, *p* > 0.05. The output of the between-subject variable showed that the main effect of color was not significant, *F*(2, 54) = 0.182, *p* = 0.835. Further generalized linear model analysis of the layout method shows that the saccade length between G1 and G2 is significantly different (*p* = 0.001), the saccade length between G1 and G3 is significantly different (*p* = 0.012), and the difference between G2 and G3 is significant (*p* = 0.012). The difference in saccade length was not significant (*p* = 0.359). The result shows that the saccade length of navigation icons with random color distribution is longer than that of navigation icons with regular color distribution, Among the navigation icons with regular color distribution, the difference in saccade length between different layouts is not significant.

**Table 12:** Results of repeated measures ANOVA on saccade length.

Factor	df	*F*	*p*
Layout	2	12.928	0.000
Color	2	0.182	0.835
Layout × color	4	1.313	0.277

*p < 0.05. *Based on the estimated marginal mean. The mean difference was significant at 0.05 level. 95% confidence interval.

Further one-way analysis of variance was conducted on the four different layouts, and the results found that the results of the saccade length analysis of the four layouts were consistent with the results of the number of fixation points and fixation time. The results show that the saccade length of the monochrome navigation icons on the mobile map is longer than that of the color-coded navigation icons, and there is no significant difference in the saccade length between the color-coded navigation icons.

### The impact of different layout methods on user experience

The subjective questionnaire takes satisfaction, ease of use, and emotional preference scores as dependent variables, and the layout of mobile map navigation icons as independent variables. One-way analysis of variance was performed on the scoring results, and the results showed that the main effect of satisfaction was significant, *F*(3, 87) = 13.911, *p* < 0.05; The main effect of availability was significant, *F*(3, 87) = 13.911, *p* < 0.05; the main effect of emotional preference was significant, *F*(3, 87) = 13.911, *p* < 0.05, This result shows that users have obvious subjective preferences for mobile map navigation icons with different layouts. Further analysis of the scoring situation shows that the average satisfaction scores given by the subjects to G0, G1, G2, and G3 are 4.233, 3.867, 6.167, and 5.833, respectively. It shows that the satisfaction of the subjects with the navigation icons of the color distribution is higher than that of the monochrome navigation icons and the navigation icons with the random color distribution. The average ease of use scores given by the subjects to G0–G3 were 3.733, 3.7, 6.167, and 5.833, which indicated that the subjects believed that the ease of use of the mobile map navigation icons with regular color distribution was better than that of the monochrome navigation icon interface and the Navigation icons with the random color distribution. The average scores of G0–G3’s emotional convenience are 3.6, 3.8, 6.1, and 5.867, which indicates that the subjects prefer the mobile map navigation icons with regular color distribution to the monochrome navigation icon interface and the layout with random color distribution Way. Table 13 shows the further within-subject comparative analysis of the four layout methods. The results show that the results of satisfaction, ease of use, and emotional preference are consistent, and the difference between G0 and G1 is not significant (*p* > 0.05). The difference between G0 and G2 was significant (*p* < 0.05), and the difference between G0 and G3 was significant (*p* < 0.05); the difference between G1 and G2 was significant (*p* < 0.05), and the difference between G1 and G3 was significant (*p* < 0.05); the difference between G2 and G3 was not significant (*p* > 0.05). This result shows that the subjects have higher scores for the navigation icons with regular color distribution, which shows that the layout mode is very important to the user experience, and the layout mode with the same color area and the larger area has the best experience.

**Table 13:** One-way ANOVA test of subjective preference with different layout methods.

	Satisfaction				Ease of use				Emotional preference			
	G0	G1	G2	G3	G0	G1	G2	G3	G0	G1	G2	G3
G0	×	0.458	0.000	0.002	×	0.458	0.000	0.002	×	0.674	0.000	0.000
G1		×	0.000	0.000		×	0.000	0.000		×	0.000	0.000
G2			×	0.499			×	0.499			×	0.623
G3				×				×				×

*p < 0.05. *Based on the estimated marginal mean. The mean difference was significant at 0.05 level. 95% confidence interval.

## Discussion

The above research results show that the layout method significantly impacts the visual search and experience of the navigation icons of the mobile map, and the eye movement data provides an objective basis for this conclusion.

### The impact of layout method on visual search efficiency

The research results show that the layout method has a significant impact on the visual search of the user’s mobile map navigation icons, and the navigation icons that use color for layout coding have the highest visual search efficiency and better user experience.

Among the navigation icons that use color for layout coding, the layout with regular color distribution and a larger area of the same color has the highest visual search efficiency and the best user experience. This result is verified not only in the data analysis of visual search time and accuracy but also in the results of eye movement data analysis. According to the feature integration theory, the user performs visual processing on the basic features in the pre-attention stage (Treisman and Gelade, 1980). In this stage, the user mainly detects the features of stimuli, and color is the most basic detection feature. Numerous studies have shown that color is the most important factor affecting users’ visual search in the pre-attention stage (Steven, 2008). Gestalt theory believes that human vision is holistic, and the visual system automatically constructs a structure for the input content and perceives the objects seen (Treisman and Gormican, 1988). People group similar-looking objects into groups. Therefore, when color is used as a variable, the user will automatically group icons of the same color. In this experiment, the layout-encoded information using color is presented in a more structured way for users to browse and understand more quickly and easily. Because in the search process, the visual hierarchy makes it easier to skip irrelevant information so that the subjects can quickly find the target and improve the efficiency of the visual search (Johnson, 2014). Wu and Chen (2009) confirmed that color grouping is one of the most important factors affecting subjects’ performance and satisfaction. The visual search efficiency of the navigation icons coded by color is significantly higher than that of the monochrome navigation icon interface. In this experiment, for a single-color icon interface, the visual system searches according to the joint features of color and shape, and the interference items are all icons in the interface. When searching for a grouped icon interface composed of two colors, the visual system can improve the classification and recognition speed of icons through color coding. Users can quickly reduce the number of interfering icons, reduce search time, and improve visual search efficiency. Therefore, when the icons of the navigation icon interface are of a single color, the color of the icons will not affect the user’s visual search efficiency. Gong et al. (2016b) confirmed this result. They pointed out that when the icon colors are inconsistent, the subject’s pre-attention processing is more, the saccade can quickly point to the target, the subject’s perceptual breadth is greater at this time, and the search efficiency is more efficient. High (Gong et al., 2016b). In this experiment, among the layout methods with the regular color distribution, the visual search efficiency of the subjects with the layout method with a larger area of the same color is higher. It is because the icons arranged in groups will form different visual search areas, and the layout with the same color area is larger. The user only needs to perform a visual search in a smaller range. Under such conditions, the user can eliminate more interference items. At this time, the subjects searched for fewer items, and the saccade distance was shorter. When subjects searched for an interface with a large color area grouping, the grouped items could be accepted or rejected together, and if accepted, the search could simply be performed within the group (Friedman-Hill and Wolfe, 1995). Item grouping reduced the number of items to be searched. For the grouping method with a small color area, although the color can help the user to classify the icons, the abnormally active color interference will distract some attention and reduce the search efficiency of the target icon. In addition, the presentation of icons with smaller color areas requires more attention span. Under such conditions, users can only adopt an extremely inefficient one-by-one search strategy, which further reduces the search efficiency of icons. Therefore, a layout with a larger color area has higher visual search efficiency.

### The impact of layout method on user experience

In this experiment, the user experience data is analyzed according to its components. The specific components are satisfaction, ease of use, and user preference.

#### The impact of layout method on satisfaction

Satisfaction is a user’s perception, feeling, and thought about a product (Rubin and Chisnell, 2017). In this experiment, when the navigation icon can better meet the user’s needs and provide better satisfaction, the user’s performance will be better when using the mobile map navigation icon. The analysis results of the subjective evaluation data show that the satisfaction of the subjects with the navigation icons with a regular color distribution is higher than that of the monochrome navigation icons and the navigation icons with a random color distribution. Zhang et al. (2019) took the user experience as the starting point and pointed out that the user experience depends on the presentation of the user interface. Spatial layout is an important presentation method of interactive interface and has an important impact on user satisfaction. Although users do not pay too much attention to good visual hierarchies, bad visual hierarchies can be highlighted in use, degrading the user experience (Susurratescape, n.d.). Compared with monochrome navigation icons and navigation icons with random color distribution, the layout of navigation icons with regular color distribution has a clearer visual hierarchy, more complete and coherent information display, and higher user satisfaction.

#### The impact of layout method on ease of use

Ease of use mainly includes efficiency and effectiveness. Efficiency is the agility of users to complete tasks accurately and completely, which can be reflected by reaction time. Effectiveness is how easy it is for users to use a product, usually measured in terms of correctness or error (Lioli and Komninos, 2016). This experiment found that the mobile map navigation icons with regular color distribution have better usability than monochrome navigation icons and navigation icons with a random color distribution. Xiong (2019) pointed out that visual interface design is an important factor affecting user usability. As we all know, layout is an important element of interface design. It carries the area division of the information. Therefore, the layout has a significant impact on the user’s usability. Navigation icons with regular color distribution are easier to help users establish a clear visual hierarchy and divide visual areas. Navigation icons with random color distribution will make the visual hierarchy of the interface cluttered, interfere with the user’s perception of information, and affect the user’s ease of use. Users cannot establish a perceptible visual system for the monochrome navigation icons, so the usability of the monochrome navigation icons is low.

#### The impact of layout method on users’ emotional preference

The user’s emotional preference depends on the user experience. The most important thing about the user experience is to maximize the humanization of the product or interface, ensure the user’s operating experience and correctness, and meet the user’s ideas and expectations. User experience design can be designed from the four dimensions of brand, usability, function and content analysis (Chen et al., 2021). Cyr et al. (2006) studied how design aesthetics affect user loyalty to a product, and the results show that good visual design has a significant impact on perceived usefulness, ease of use, and pleasure. By optimizing the design of these factors, user loyalty can be improved. In this experiment, through the analysis of the behavior data of the subjects, it was found that the navigation icons with regular color distribution had shorter operation times and higher accuracy. The result shows that the navigation icons with regular color distribution are more effective, and the subjects are more efficient in completing the operational tasks. Moreover, the subjects were more satisfied with the mobile map navigation icons in this layout, which indicated that the layout had the best usability, so the user preference for navigation icons with regular color distribution was higher.

### Limitations and future work

There are several limitations. First, the experiment uses the computer interface instead of the mobile interface and uses the keyboard to replace the touch screen operation. Although this experimental paradigm can accurately record the experimental data, it is different from the real use scene. In subsequent studies, experiments can be performed using real mobile devices.

Second, the eye-tracking data in this experiment were collected by the EyeLink fixed eye-tracking instrument. During the experiment, although the data collection of the eye-tracking instrument with the fixed head was accurate, it could not restore the actual usage of mobile devices. In future experiments, head-mounted eye-tracking equipment can be used to make the user’s experimental process more realistic.

Third, the research objects of this study are undergraduates and postgraduates. This group may have certain common characteristics and cannot represent all user groups, and the sample is slightly insufficient. In the follow-up research, the sample can be expanded to make the experimental data more objective and accurate.

Fourth, the visual search experimental paradigm in this study is to present the target icon first, then the interference icon interface, and ask the subjects to find the target icon in the interference icon interface. There are some differences between the real mobile information map usage scenarios and this experimental paradigm. The experimental paradigm can be improved in subsequent studies.

## Conclusion

This study firstly analyzes the navigation icons of the mobile map and designs the navigation icons based on color coding and spatial layout, and then conducts an experimental study on the visual search efficiency of the mobile map navigation icons based on color coding and spatial layout. The main conclusions are as follows: First, the layout of the mobile map navigation icons has a significant impact on the user’s visual search. Among them, the navigation icons that use color for layout coding have the highest visual search efficiency and better user experience. Second, among the navigation icons that use color for layout coding, the layout with regular color distribution and a larger area of the same color has the highest visual search efficiency for users and the best user experience. Third, the visual search efficiency of navigation icons using color for layout coding is significantly higher than that of monochrome navigation icons. The research results have certain practical significance. This research provides design guidance for the design of mobile phone map navigation icons, and provides an important theoretical basis for the design of human-computer interaction interface and user experience. In the future, we can further combine eye tracking technology to conduct in-depth research on icon visual search strategies, and further explore the impact of different methods or number of layouts on users’ visual search.

## Data availability statement

The original contributions presented in the study are included in the article/supplementary material, further inquiries can be directed to the corresponding authors.

## Ethics statement

Written informed consent was obtained from the individual(s) for the publication of any potentially identifiable images or data included in this article.

## Author contributions

MZ conceived and conducted experiments and wrote most of the manuscript. YG proposed research topics and invited experimental subjects. RD made article fixes. All authors contributed to the article and approved the submitted version.

## Funding

This research was funded by the National Key R&D Program (2017YFB1002605) and Ningbo University Humanities and Social Sciences Special Project/Cultivation Project (XPYB18005).

## Conflict of interest

The authors declare that the research was conducted in the absence of any commercial or financial relationships that could be construed as a potential conflict of interest.

## Publisher’s note

All claims expressed in this article are solely those of the authors and do not necessarily represent those of their affiliated organizations, or those of the publisher, the editors and the reviewers. Any product that may be evaluated in this article, or claim that may be made by its manufacturer, is not guaranteed or endorsed by the publisher.
